# A Case Report on Chikungunya Virus-Associated Encephalomyelitis

**DOI:** 10.1155/2018/8904753

**Published:** 2018-07-10

**Authors:** Harsh Khatri, Heli Shah, Dhara Roy, Kaushalendra Mani Tripathi

**Affiliations:** Department of Medicine, V.S. Hospital, Smt. NHL Municipal Medical College, Ahmedabad, India

## Abstract

Chikungunya is a rerising alphavirus infection that has resulted from enhanced vector competence. Alphaviruses are divided into arthritogenic viruses (old world) and encephalitogenic viruses (new world) including equine encephalitis viruses. Chikungunya is a dengue-like illness characterized by acute febrile polyarthralgia, arthritis, malaise, body ache, rash, headache, and nausea. The illness is self-limiting. The neurological manifestations are uncommon and incorporate meningoencephalitis, myelitis, Guillain–Barre syndrome, cranial nerve palsies, myelopathy, and neuropathy; MRI abnormalities in patients with encephalopathy from India have been reported in the form of multiple punctuate white matter lesions that are more prominent on diffusion-weighted MRI than on T2 or T1. Here, we present an intriguing case of chikungunya encephalomyelitis who presented to our tertiary care hospital with quadriparesis and urinary retention. He was treated with 5 doses of intravenous immunoglobulin along with supportive care with which he showed partial recovery.

## 1. Introduction

Chikungunya is a reemerging alphavirus infection that has resulted from enhanced vector competence. Alphaviruses having single-stranded RNA genome belong to Togaviridae family of viruses and are divided into arthritogenic viruses (old world) and encephalitogenic viruses (new world) including equine encephalitis viruses. It is transmitted by the mosquitoes of *Aedes* genus amongst which *Aedes aegypti* being the principal vector in earlier times now viral host range includes *A. albopictus* as well [1].

From the first isolation of virus in 1952-53 from Tanzania [2], it has spread in tropical countries to such an extent that 2006 outbreak in India reported 1.5 million cases with *A. Aegypti* implicated as the vector. Chikungunya virus was first isolated in Calcutta, India, in 1963, with several reported outbreaks in India since then. In the year 2016, clinically suspected cases of chikungunya in India were 64057 [3].

Neurological complications of chikungunya virus (CHIKV) infection are infrequent, but they can include a spectrum of different neurological manifestations because of neurotropism demonstrated by this virus. Herein, we describe an adult case of CHIKV-associated central nervous system (CNS) disease.

## 2. Case Report

### 2.1. History and Examination

A 64-year-old male patient, resident of Vadodara city, Gujarat state located in the western India, who is a known case of diabetes mellitus II, hypothyroidism, and ocular myasthenia gravis since 3 years presented to our tertiary care hospital named Sheth VS General hospital located in Ahmedabad city (Gujarat state) with 8 days history of acute fever, malaise, generalized rash, and multiple joint pains. He complained of an acute onset of sensorimotor quadriparesis and urinary retention since 7 days which was followed 1 day later by H/O altered sensorium. He had no history of headache, vomiting, seizure, dimness of vision, double vision, dysphagia, change in voice, and neck/back pain. The patient had already received methyl prednisolone injection pulse therapy 1 g each for 5 days before presenting to our institute to which he responded partially in form of improved level of consciousness. The patient had no recent history of travelling outside the state of Gujarat or India.

#### 2.1.1. On Presentation: CNS Examination

The patient was conscious oriented following verbal command and had no neck rigidity. Cranial nerves: left eye ptosis+; no facial/neck flexor weakness; and mixed dysarthria+.

#### 2.1.2. On Motor Examination

Nutrition-no undue wasting or hypertrophy; tone: spastic both upper limbs (UL) with flaccid both lower limbs; power (according to the MRC scale): 4/5 in both UL and 1/5 in both LL; with B/L hand grip weakness and B/L dorsiflexor weakness;

Deep tendon reflexes (DTRs) were +3 in both UL with B/L pectoralis reflex and jaw jerk+; DTRs were absent in both lower limbs (LL), and planters were absent.

#### 2.1.3. Sensory Examination

Impaired joint, position, and vibration sensations up to metatarsophalangeal joints in both the lower limbs and up to metacarpophalangeal joints in both the upper limbs were noted with normal cerebellar examination.

### 2.2. Investigations

#### 2.2.1. Routine Investigations

Including complete blood count, random blood sugar, renal and liver function tests, and serum electrolytes were within normal limits except for slightly raised WBC to 14500/*µ*L, CRP 16, and CPK total 84. Serum CHIKV IgM detection by ELISA came positive. Serum chikungunya real-time PCR, which is a qualitative test of chikungunya RNA by a standard procedure, was positive. RNA was extracted using the QIAamp Viral RNA Mini kit (Qiagen). One-step RT-PCR was performed using the access quick RT-PCR kit (Promega), in accordance with the manufacturer's protocol, employing primer pairs targeting the E1 gene designed from the nucleotide sequence of the reference S27 strain (GenBank accession number AF490259; CK 13, TTA CAT CAC GTG CGA TA C; CK-14, CTT TC TCT CAG GG TGC GAC TTT). The amplification was performed in a 50 *µ*L total reaction volume with the Promega Access Quick One-Step RT-PCR kit, 50 pmol of each forward and reverse primer, and 2 *µ*L of extracted viral RNA, in accordance with the manufacturer's instructions.

Electroencephalogram (EEG) showed generalized bilaterally symmetrical diffuse theta-delta slowing suggestive of diffuse encephalopathy. His electrophysiology study showed distal symmetrical sensory motor axonal polyneuropathy more in the lower limbs than the upper limbs. CSF routine micro: protein, 96 mg/dL, glucose, 60 mg/dL, and total cells, 140 with lymphocytic pleocytosis with negative Gram and ZN stain. CSF viral panel done by PCR was negative for neurotropic viruses. This included pan-flavivirus RNA, pan-enterovirus RNA, pan-paramyxovirus RNA detection and pan-herpes DNA detection. Patient's sample was positive for ANA (antinuclear antibody) by immunofluorescence with a dilution of 1 : 100 and 1 : 200, a homogeneous pattern, and +1 intensity. On further workup, ANA profile by immunoDOT was negative. Anti-aquaporin-4 Ab became negative. Serum and CSF investigations are depicted in Tables 1 and 2.

#### 2.2.2. MRI Brain (P + C) with Whole Spine Screen

**Figure 1:**
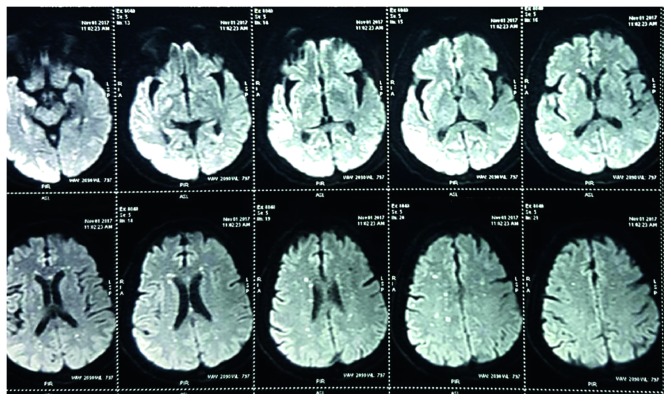
Multiple tiny areas with restricted diffusion in subcortical and periventricular white matter on axial DWI brain.

**Figure 2:**
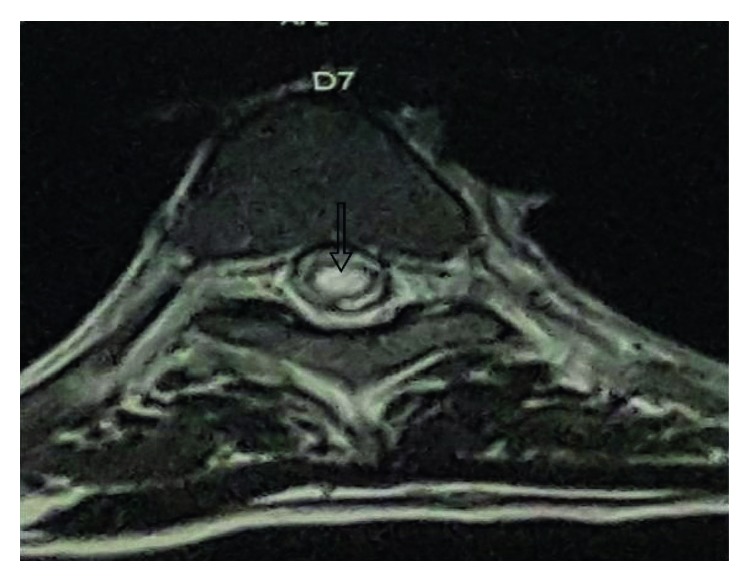
Axial T2W sagittal section showing D7 intramedullary hyperintensity.

**Figure 3:**
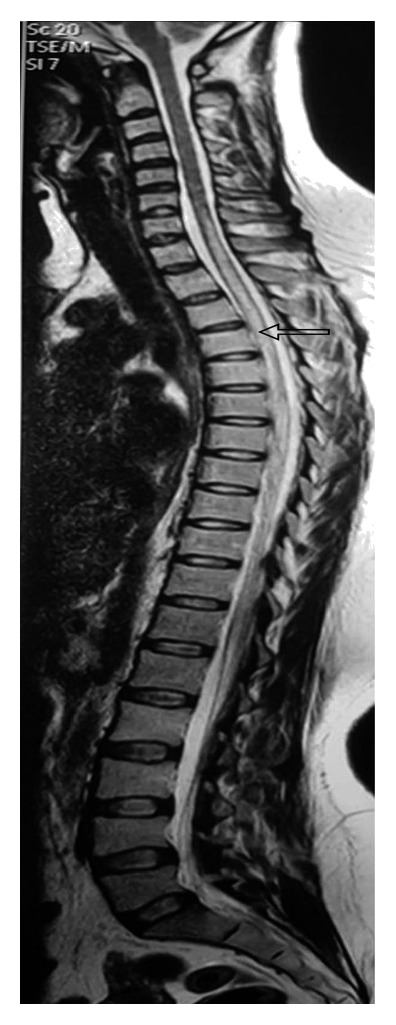
T2W sagittal image showing intramedullary hyperintensity extending from C7–D9 segment (long-segment myelitis).

Multiple tiny altered signal intensity foci with restricted diffusion seen in subcortical and periventricular white matter of bilateral (B/L) cerebral hemispheres, corpus callosum, and b/L corona radiata with normal MR angiogram are shown in Figure 1. Axial T2W sagittal section showing D7 intramedullary hyperintensity is shown in Figure 2. Patchy areas of T2W hyperintensity in the cervicodorsal cord predominantly from the C7 to D9 level without significant postcontrast enhancement are shown in Figure 3. These findings were consistent with features of encephalomyelitis.

### 2.3. Management and Outcome

Our patient was managed with IV immunoglobulin (IgG) in a total dose of 140 grams as well as supportive treatment for his comorbidities. He was discharged in a stable hemodynamic condition with power 5/5 in both the UL and 3/5 in both the LL. The patient was advised supportive treatment and neurorehabilitation in form of active physiotherapy.

#### 2.3.1. Follow-Up after 1.5 Months

The patient responded well; his power improved; he was able to walk with one stick support with a normal bladder function with a current modified Rankin scale (mRS) of 4. No radiological investigations were done in follow-up.

## 3. Discussion

The neurological manifestations associated with chikungunya are uncommon and variable. Various manifestations like meningoencephalitis, myelitis, Guillain–Barre syndrome, cranial nerve palsies, encephalomyeloradiculitis, and neuropathy have been reported [1, 2].

In this unique case of chikungunya encephalomyelitis, diagnosis was based on positive concordance between clinical presentation, serology, CSF, and neuroimaging features. Other differential diagnoses were ruled out by thorough investigations.

MRI brain showed bilateral multiple tiny punctuate white matter lesions with increased signals on DWI (diffusion-weighted sequence), which is quite a characteristic of early chikungunya encephalitis as demonstrated by case reports in Brazil by Pereira et al. [4], in India by Ganesan et al. [5], in southern Thailand by Chusri et al. [6], and again in Brazil in a neonate etiology being vertical transmission by Bandeira et al. [7]. Restricted diffusion precedes signal abnormalities seen on fluid-attenuated inversion recovery (FLAIR) images and is also known to resolve earlier than the FLAIR signal abnormalities during the recovery period [4]. MRI spine showed T2W intramedullary hyperintensities in the cervicodorsal region with long-segment transverse myelitis. These changes of encephalomyelitis were similar to the case report by Taraphdar et al. [8] in West Bengal.

The pathophysiological changes leading to such characteristic neuroimaging findings include bilateral white matter lesions with restricted diffusion which is implicated to cytotoxic edema secondary to plasma leakage from capillaries and venules. Such changes can also occur in vasculitis or acute demyelination induced by sensitization to viral antigen and microglial activation [4].

## 4. Conclusion

Chikungunya-related CNS complications should be searched for in patients from the endemic areas with prominent neurological features on examination. Radio imaging along with serology and CSF help to confirms the diagnosis. This case report does highlight the importance of clinicoradiologic findings in patients with encephalomyelitis following chikungunya infection.

## Figures and Tables

**Table 1:** 

Serum investigations	Patient value	Reference value
Haemoglobin	11.5 G%	13–17 G%
White blood cell count	14500/*µ*L	4000–10000/*µ*L
C-reactive protein	16 mg/L	0–10 mg/L
Total CPK	84 U/L	52–336 U/L for male
Anti-aquaporin-4 (NMO-IgG antibody)	Not detected	—
Serum chikungunya real-time PCR	CHIKV detected	—
Serum CHIKV IgM by ELISA	Positive for CHIKV	—

NMO Ab was negative.

**Table 2:** 

CSF investigations	Patient value	Reference value
Protein	96 mg/dL	15–50 mg/dL
Glucose	60 mg/dL	45–100 mg/dL
Total white blood cells	140/cmm (100% lymphocytes)	<08/cmm
Pan-neurotropic viral panel	Negative (see text)	—
